# Metabolic Engineering of Isoflavonoid Biosynthesis by Expressing *Glycine max Isoflavone Synthase* in *Allium cepa* L. for Genistein Production

**DOI:** 10.3390/plants10010052

**Published:** 2020-12-29

**Authors:** Ashwini Malla, Balamurugan Shanmugaraj, Balamurugan Srinivasan, Ashutosh Sharma, Sathishkumar Ramalingam

**Affiliations:** 1 Plant Genetic Engineering Laboratory, Department of Biotechnology, Bharathiar University, Coimbatore, Tamil Nadu 641 046, India; malla164_ashwini@yahoo.co.in (A.M.); balasbm17@gmail.com (B.S.); balasvm@gmail.com (B.S.); 2 Tecnologico de Monterrey, School of Engineering and Sciences, Centre of Bioengineering, Campus Queretaro, Av. Epigmenio González No. 500, Fracc. San Pablo, Queretaro 76130, Mexico

**Keywords:** genistein, isoflavone synthase, isoflavonoids, micropropagation, naringenin, onion, transformation

## Abstract

Isoflavonoids, the diverse group of secondary metabolites derived from the phenylpropanoid pathway, are distributed predominantly in leguminous plants and play a vital role in promoting human health. Genetic engineering of the metabolite synthesis pathway has turned out to be an attractive approach for the production of various secondary metabolites. In our study, we attempted to produce the isoflavone genistein, a well-known health-promoting metabolite, in *Allium cepa* L. (onion) by introducing *Glycine max Isoflavone synthase* (*GmIFS*). The *GmIFS* gene was cloned into the pEarleyGate 102 HA vector and transformed into onion by *Agrobacterium*-mediated and biolistic methods. The presence of *GmIFS* in transgenic onion was confirmed by PCR, dot blot, and Southern hybridization. Analysis of the transgenic onion calli lines demonstrated that the expression of the *GmIFS* gene led to the production of isoflavone genistein in *in vitro* tissues. The biolistic stable transformed calli with transformation efficiency of 73% (62.65 nM/g FW) accumulated more genistein than the *Agrobacterium* stable transformed calli with transformation efficiency of 56% (42.5 nM/g FW). Overall, heterologous gene expression of *GmIFS* was demonstrated by modifying the secondary metabolite pathway in onion tissues for the production of isoflavone genistein that can boost up human health with its health-promoting properties.

## 1. Introduction

*Allium cepa* L., commonly known as onion, is one of the most important commercial condiment vegetables grown and consumed all over the world. In pharmacologic and *in vitro* studies, onion and its extract, alone and in combination with other products, have shown antioxidant, anticancer, antimicrobial, asthma, cardiovascular, anti-carcinogenic, anti-platelet, anti-thrombotic, anti-asthmatic, anti-diabetic, fibrinolytic, hypocholesterolemic, antibiotic properties, and hemostatic effects [1,2,3,4,5]. Although extensive studies and copious literature is available on onion and its curative effects, more research has to be delved into to comprehend and enhance the therapeutic and salutary effects of onion bulbs by genetic modifications.

The use of plants for medicinal purposes dates back thousands of years but genetic engineering of plants to produce desired biopharmaceuticals is much more recent. As there is a demand for increased biopharmaceuticals, it would be wise to ensure that they will be available in significantly larger amounts on a cost-effective basis. Naringenin, a flavonone intermediate is pervasive in plants acting as a substrate for production of isoflavonoids in the presence of *isoflavone synthase* (*IFS*). In the members of *Leguminaceae* family, *IFS* is a key enzyme that regulates the synthesis of isoflavonoids genistein and daidzein by redirection of flavonone intermediates, naringenin, and liquiritigenin, respectively in the phenylpropanoid pathway [6,7]. The heterologous expression of *IFS* for genistein biosynthesis was studied in *Arabidopsis thaliana*, *Nicotiana benthamiana*, *Solanum lycopersicum* (tomato), *Oryza sativa* (rice), *Petunia alba*, *Lactuca sativa* (lettuce) [6,8,9,10].

Hence, this study aims to produce the isoflavonoid genistein in onion by expressing the *Glycine max isoflavone synthase* (*GmIFS*) under the control of cauliflower mosaic virus 35S (CaMV 35S) constitutive promoter. The transgene was transformed into *in vitro* grown onion tissues by *Agrobacterium* and biolistic-mediated transformation and its presence in transformants was confirmed by polymerase chain reaction (PCR). Further, the genistein production in transgenic onion was demonstrated. To the best of our knowledge, this is the first report on the production of isoflavonoid genistein in transgenic onion.

## 2. Results

### 2.1. In Vitro Propagation and Genetic Transformation of Onion Calli

The *in vitro* growth conditions for onion micropropagation was optimized and followed as described in Malla et al., 2015 [11]. Embryogenic callus formation was induced on MS+B5 medium supplemented with 1.0 mg L^−1^ picloram. Bulb induction was observed on MS+B5 medium containing 2.0 mg L^−1^ BAP. Onion calli were regenerated on MS media supplemented with two cytokinins and one auxin (BAP, KIN (0.5 mg L^−1^) and NAA (0.1 mg L^−1^)).

**Figure 1:**
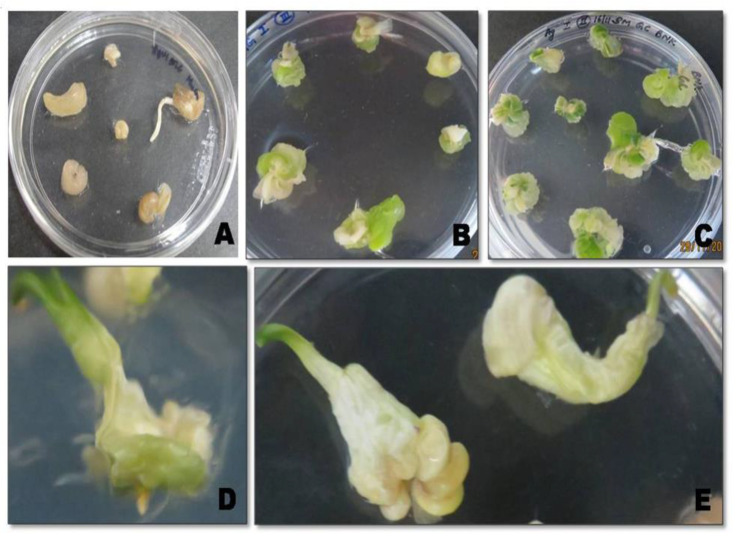
*Agrobacterium*-mediated transformation in onion calli. (**A**) Wild type calli on selection media; (**B**,**C**) Transformed calli on selection media (MS+B5 + 1.0 mg L^−1^ picloram + 20 mg L^−1^ glufosinate-ammonium + 500 mg L^−1^ cefotaxime; (**C**) Transformed calli after four rounds of selection; (**D**) Transformed callus on regeneration medium (MS + 0.5 mg L^−1^ KIN + 0.5 mg L^−1^ BAP + 0.1 mg L^−1^ NAA + 20 mg L^−1^ glufosinate-ammonium); (**E**) Transformed callus on bulb initiation medium (MS + B5 + 2.0 mg L^−1^ BAP).

*Agrobacterium*-mediated DNA delivery has been successfully obtained for bellary onion variety. *Agrobacterium* AGL1 strain harboring the pEarleyGate 102 HA plasmid with *GmIFS*, under the CaMV 35S promoter was used for genetic transformation. After the co-cultivation period of 3 days, onion calli were placed on selection media (MS+B5 + 1.0 mg L^−1^ picloram + 20 mg L^−1^ glufosinate- ammonium + 500 mg L^−1^ cefotaxime) to select the transformants. Shoots were induced in regeneration media after 4 weeks of selection from the callus. The growth of the shoots in selection media was an indication of integration of the *GmIFS* in the transgenic onion calli (Figure 1).

**Figure 2:**
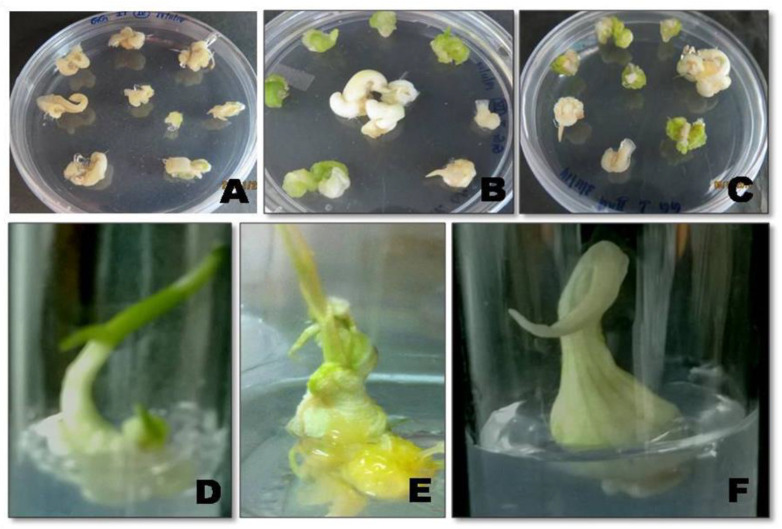
Biolistic/gene gun-mediated transformation in onion calli. (**A**) Wild type calli on selection media; (**B**,**C**) Transformed calli on selection media (MS+B5 +1.0 mg L^−1^ picloram + 20 mg L^−1^ glufosinate-ammonium; (**C**) Transformed calli after four rounds of selection; (**D**,**E**) Transformed callus on regeneration medium (MS + 0.5 mg L^−1^ KIN + 0.5 mg L^−1^ BAP + 0.1 mg L^−1^ NAA + 20 mg L^−1^ glufosinate-ammonium); (**F**) Transformed callus on bulb initiation medium (MS+B5 + 2.0 mg L^−1^ BAP).

Explants for biolistic-mediated genetic transformation were *in vitro* grown onion calli. After bombarding the explants at 1300 psi, they were placed on the pre-culture media for 3 days, to allow for expression of the integrated gene. Subsequently, they were placed in selection media (MS+B5 + 1.0 mg L^−1^ picloram + 20 mg L^−1^ glufosinate-ammonium) to screen the transformants. The calli were well differentiated. The individual calli were separated and placed into regeneration media (MS + 0.5 mg L^−1^ KIN + 0.5 mg L^−1^ BAP + 0.1 mg L^−1^ NAA + 20 mg L^−1^ glufosinate-ammonium). Once a considerable amount of shooting was obtained, they were inoculated on bulb initiation medium MS+B5 supplemented with BAP (Figure 2).

### 2.2. Molecular Characterization of Transgenic Onion Calli

**Figure 3:**
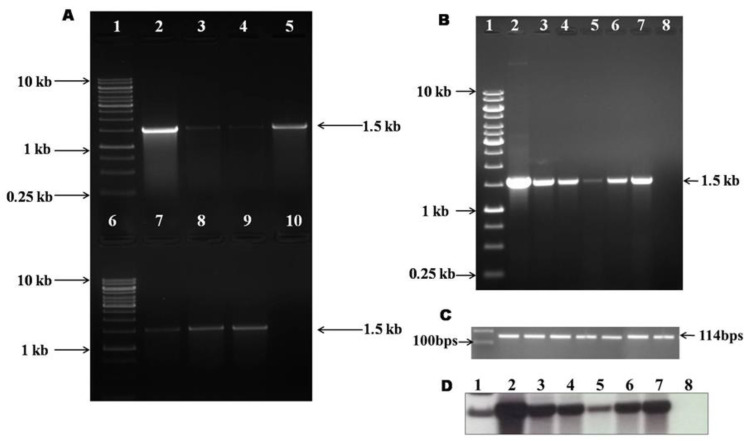
Confirmation of transgene integration. (**A**) PCR confirmation of *GmIFS* expression in *in vitro* grown onion calli. Lane 1,6: 1 kb DNA marker, Lane 2,7: Positive control (plasmid), Lane 3–5: *Agrobacterium*-mediated transformed callus, Lane 8–9: Biolistic-mediated transformed callus, Lane 10: Negative control (wild type callus); (**B**) Reverse transcriptase PCR showing the expression of *GmIFS* in stable transgenic callus; (**C**) β-Actin internal control; (**D**) Confirmation of gene integration by Southern hybridization. Lane 1: 1 kb DNA marker, Lane 2: Positive control (plasmid), Lane 3–5: *Agrobacterium* transformed callus, Lane 6–7: Gene gun transformed callus, Lane 8: Negative control (wild type callus).

The putative transgenic calli were used for DNA isolation using Macherey-Nagel Plant DNA isolation kit to screen the presence of *GmIFS*, using gene-specific primers. The expected amplicon (1.5 kb) was observed in the *Agrobacterium* transformed lines and biolistic transformed lines (Figure 3A). The stable expression of *GmIFS* in the onion calli was analyzed by reverse transcriptase PCR. From the isolated RNA, cDNA was synthesized and it was used as the template for the amplification of *GmIFS* using gene-specific primers. The *GmIFS* gene expression under CaMV 35S promoter was confirmed by expected amplicon size of 1.5 kb on agarose gel electrophoresis (Figure 3B). The *β-Actin* (~114 bp) was used as an internal control (Figure 3C).

To check the integration of *GmIFS* in the transgenic onion callus, the genomic DNA was further analyzed by dot blot hybridization. The development of purple colored spot confirmed the integration of *GmIFS* gene in the transgenic onion calli/bulbs (Supplementary Figure S1). Further, the genomic DNA isolated from *Agrobacterium* and biolistic transformed onion calli were subjected to Southern hybridization. Southern blot analysis confirmed the integration of *GmIFS* gene in the transgenic calli lines. The hybridization signal corresponds to the length of *GmIFS* that is observed in the transgenic onion calli, while there was no signal observed in the wild type control callus (Figure 3D).

### 2.3. Metabolite HPLC Analysis

**Figure 4:**
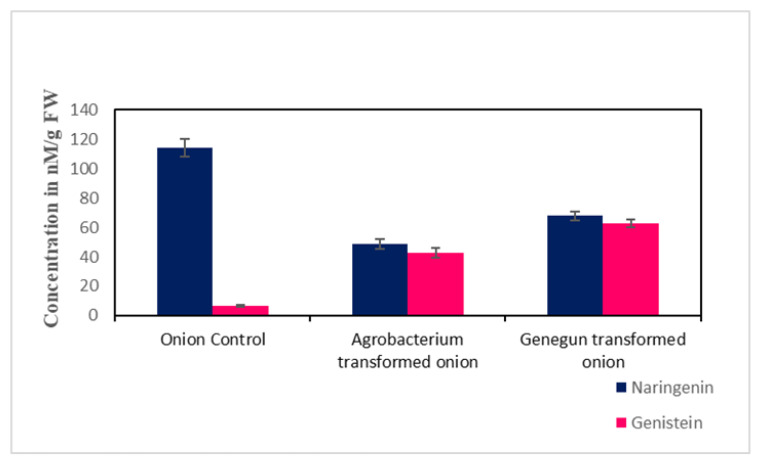
Quantification of naringenin and genistein in control and transformed *in vitro* grown onion lines. The HPLC analysis showed the presence of naringenin in control (wild type onion) and genistein accumulation was observed in *Agrobacterium* and biolistic transformed onion lines.

Reversed phase HPLC methods with C_18_ or C_8_ columns are most commonly used for the separation of naturally occurring flavonoid glucosides and their aglycones in crude plant extracts, food products, and biological fluids using the mobile phases consisting of acetonitrile/water as well as methanol/water. The column temperature (20–45 °C), solvent system, sample preparation were important factors for improving separation of vital compounds in the samples to give symmetrical and sharp peaks [12]. Well-resolved peaks were obtained for both naringenin and genistein standards when 30% acetonitrile and 80% methanol, respectively, were used as eluents. Onion calli transformed with *GmIFS* showed less concentration of naringenin compared to the wild type onion callus (48.75 and 68 nM/g FW, respectively) (Figure 4).

Quantification analysis showed that all transgenic lines transformed with *GmIFS* accumulated comparable amounts of genistein. The wild type control plants showed negligible accumulation of genistein in comparison with the transgenic lines. The biolistic transformed onion calli (62.65 nM/g FW) showed higher amounts of genistein synthesis than the *Agrobacterium*-mediated transformed onion callus (42.5 nM/g FW) (Figure 4).

## 3. Discussion

Isoflavonoids, human health-promoting bioactive molecules with multiple functions, are ubiquitously present in *Leguminaceae* plant members. Onion is one of the important edible plants with a rich nutritional value and powerful health-promoting properties [13]. We have expressed *GmIFS* gene in onion that led to the production of isoflavonoid genistein in transgenic plants using endogenous naringenin as the substrate. *Agrobacterium*-mediated DNA delivery has been successfully developed for bellary onion variety. One of the transformation procedures was based on *Agrobacterium tumefaciens*, using two-to-four-week-old callus as a target tissue. Various factors influencing *Agrobacterium*-mediated transient expression of *uid*A were intensely studied in rice and wheat [14,15]. Optimization of the co-cultivation time, co-cultivation medium, and selection medium proved to be of considerable importance. Activation of the *A. tumefaciens* virulence (*vir*) genes was also a critical step in transformation because they have to be induced by specific phenolic compounds such as acetosyringone [15,16,17]. Therefore, we added 150 μM acetosyringone both to the *Agrobacterium* liquid culture medium and to the co-cultivation medium. The other for successful transformation might be due to the chopping of the callus just before the co-cultivation with the *Agrobacterium* and the subsequent selection phase with glufosinate-ammonium. The effect of specific *Agrobacterium* strains and vectors in transformation process varies as it differed by using ‘super-virulent’ strains, which had been emphasized in previous reports [18,19,20]. In our study, *A.tumefaciens* strain AGL1 was chosen for transformation in onion, as it has been found to be the most efficient strain for monocot transformation [21]. The AGL1 strain showed higher transformation frequencies in *Triticum aestivum* L. (wheat), *Zea mays* L. (maize), *Solanum chacoense* L. and *Solanum tuberosum* L. (potatoes) [22,23,24].

Genistein contents achieved in our study were comparable with that in a previous report, but seemed to be too low for applications such as dietary disease prevention, considering the current recommended doses [25]. Previously, overexpressing an *IFS* gene alone generally resulted in relatively low isoflavone yields in transgenic plants. For example, transgenic *Arabidopsis* accumulated only up to 5.4 nM/g FW of genistein in leaves [26], whereas transgenic *Petunia* accumulated similar amounts of genistein in leaves (3.4 nM/g FW) and petals (7.4 nM/g FW) [27].

Manipulations of endogenous flavonoid pathways have been used as an approach to elevate isoflavone production in transgenic plants. Naringenin is a common precursor for diverse classes of flavonoids, including isoflavones, flavones, flavonols, and anthocyanins. Few studies demonstrated that isoflavones usually exist as glucosyl and malonyl-glucosyl conjugates, which are the inactive forms and should be acid hydrolyzed to their bioactive aglycone forms for HPLC detection [28]. Reports showed that the biosynthesized genistein in transgenic rice, tobacco, *Arabidopsis*, and maize BMS cell line was also found to be present in conjugated forms suggesting that the varied host enzymes may be responsible for these types of modifications in tissues that do not naturally encounter genistein [8,26]. However, in the present study, the biosynthesized genistein was detected by HPLC, confirming its bioactive form in transgenic onion tissues.

In summary, metabolic engineering in plants holds a promising way of producing isoflavonoids that have high nutrient and therapeutic properties. In this study, we have transformed the *GmIFS* gene in onion by *Agrobacterium* and biolistic-mediated transformation. Further, the *in vitro* grown transgenic onion was confirmed and the production of genistein from the endogenous substrate naringenin was demonstrated. Taken together, our results showed that isoflavonoids can be produced in non-leguminous crops by expressing *IFS* gene. To the best of our knowledge, this is the first report showing the production of isoflavonoids in transgenic onion. A final aspect of interest to consider in the future is to increase the substrate availability of genistein by overexpressing other critical pathway enzyme genes together with *IFS*, which might further improve the isoflavone production in onion or other edible crop plants.

## 4. Materials and Methods

### 4.1. Construction of Plant Expression Vector

*GmIFS* cDNA (Genbank accession number AF195798) was kindly provided by Dr. Oliver Yu and Dr. Brian Mc Gonigle, Donald Danforth Plant Science Center, St. Louis, MO, USA. The *GmIFS* gene was cloned into plant expression vector pEarleyGate 102 HA by gateway cloning technology (Invitrogen, Carlsbad, CA, USA). The *GmIFS* gene was amplified by PCR and purified PCR product was cloned into entry vector pDONR/Zeo. The reaction product was then transformed into DH5α strain of *E. coli* by heat shock method and plated on low salt LB media supplemented with Zeocin (50 mg L^−1^). Transformed colonies were inoculated in sterile low salt LB broth with zeocin (50 mg L^−1^) and incubated in orbital shaker at 37 °C overnight. The plasmid was isolated from the grown colonies as per the manufacturer’s protocol (Macherey-Nagel Plasmid DNA Isolation Kit, Duren, Germany) and confirmed by PCR with gene-specific primers and restriction digestion. The *GmIFS* insert in the entry vector was then moved to the gateway destination vector pEarleyGate 102 HA and then transformed into *E. coli* by heat shock method and plated on LB media supplemented with kanamycin (75 mg L^−1^). The transformed colonies in the destination vector were confirmed by gene-specific PCR and restriction mapping. Subsequently, the *GmIFS* transformation vector was transformed to *Agrobacterium tumefaciens* strain AGL1 by electroporation.

### 4.2. In Vitro Culture and Agrobacterium-Mediated Genetic Transformation of Onion

The bellary cultivar onion bulbs were obtained from the local vegetable markets in Coimbatore, Tamil Nadu. The bulbs were surface sterilized and inoculated on MS media fortified with plant growth regulators for callus induction, shoot regeneration, and bulb initiation as per *in vitro* methods described in Malla et al., 2015 [11].

*A. tumefaciens* AGL1 strain with pEarleyGate 102 HA plasmid harboring *GmIFS* gene under the control of CaMV 35S promoter, was used for genetic transformation. The *Agrobacterium* culture was grown in YENB medium supplemented with 60 mg L^−1^ rifampicin and 75 mg L^−1^ kanamycin and the cultures were maintained at 28 °C in an orbital shaker. Overnight grown culture of *Agrobacterium* (OD_600_: 0.6–0.8) harboring *GmIFS* was pelleted at 8000 rpm for 10 min and the cells were resuspended in liquid MS solution, and 150 μM acetosyringone was added. The 14–21 days old chopped calli of onion were immersed in this solution for 20 min, followed by washing in water with 500 mg L^−1^ cefotaxime for 10 min. The explants were blot dried completely on sterile filter paper for 20–30 min and inoculated in co-cultivation media containing acetosyringone, glufosinate-ammonium (20 mg L^−1^), cefotaxime (500 mg L^−1^) and incubated for 3 days in the dark. After the co-cultivation period, the calli were inoculated on callus induction medium with 1.0 mg L^−1^ picloram, glufosinate-ammonium (20 mg L^−1^), and cefotaxime (500 mg L^−1^) for selection. After 4 rounds of selection, with subculture on every 7th day, the healthy transformed calli were inoculated on regeneration media containing MS + 0.5 mg L^−1^ KIN + 0.5 mg L^−1^ BAP + 0.1 mg L^−1^ NAA + 20 mg L^−1^ glufosinate-ammonium. After regeneration of shoot(s), it was transferred onto bulb initiation medium supplemented with MS+B5 + 2.0 mg L^−1^ BAP.

### 4.3. Biolistic-Mediated Genetic Transformation

Particle bombardment was performed using biolistic equipment from Gene Pro He, Hyderabad, India. The surface sterilized tungsten, 10 μg of pEarleyGate 102 HA *GmIFS* plasmid DNA, 2.5 M CaCl_2_, freshly prepared 100 mM spermidine were vortexed briefly. The above mixture was placed on ice to precipitate the tungsten mixture at the bottom of tube. Plasmid DNA-tungsten mixture was suspended in filter holder. The explants (onion calli) were placed in sliding tray. The helium source was opened until the pressure reached 1300 psi and the vacuum pump was opened and stopped once the vacuum reached 600 psi. The gene gun was turned on and shot. The gene-gun shot onion calli were inoculated on selection media supplemented with 20 mg L^−1^ glufosinate-ammonium. After 4 rounds of selection, the same procedure mentioned in 4.2 was followed to regenerate the shoots and induce bulb initiation for biolistic transformed onion calli.

### 4.4. Screening of Transformants

For PCR confirmation of putative transformants, genomic DNA was extracted from callus/bulbing tissues by following the manufacturer’s instructions given in the Macherey Nagel Plant DNA isolation kit, Germany. For gene expression analysis, total RNA was extracted from callus/bulbing tissues using Trizol method (Invitrogen, Carlsbad, CA, USA). RNA samples were DNase-I treated (Invitrogen, Carlsbad, CA, USA) and reverse-transcribed by M-MLV reverse transcriptase (Sigma Aldrich, St. Louis, MO, USA). The synthesized cDNA was used as template and the PCR was performed using the gene-specific forward primer (5′ ATG TTG CTG GAA CTT GCA CTT GGT 3′) and reverse primer (5′ TTA AGA AAG GAG TTT AGA TGC AAC GCC 3′) by using EmeraldAmp MAX PCR master mix (Takara Bio Inc., Shiga, Japan). PCR cycling conditions were as follows: Initial denaturation (94 °C for 5 min), followed by 25 cycles of denaturation (94 °C for 30 s), annealing (56 °C for 30 s), and extension (72 °C for 45 s), followed by a final extension step at 72 °C for 10 min. The DNA isolated from both *Agrobacterium* and biolistic transformed calli were used for dot blot analysis and southern hybridization. For dot blot analysis, 0.5 μg of heat-denatured genomic DNA from the transgenic lines were spotted on the nitrocellulose membrane. Biotinylated DNA probe complementary to the *GmIFS* was used that bound to the *GmIFS* of genomic DNA of the transgenic lines. Biotin bound to streptavidin conjugated with alkaline phosphatase, was subsequently detected by chromogenic substrate. For Southern hybridization, the genomic DNA from the transgenic lines was digested with the restriction enzymes *Sac*I and *Nco*I, which flank the *GmIFS* gene in the T-DNA region. Biotinylated DNA probe complementary to the *GmIFS* was used to bind to the *GmIFS* sequence in genomic DNA. Biotin bound to streptavidin conjugated with alkaline phosphatase was subsequently detected by chromogenic substrate.

### 4.5. Metabolite Analysis of Transgenic Onion Extracts

Authentic standards of naringenin and genistein (Sigma Aldrich, St. Louis, MO, USA) were used for compound identification and quantification. Approximately 0.5 g of tissues was extracted in 100% methanol for HPLC analysis. The extracts were dried using a vacuum concentrator (Concentrator 5301, Eppendorf, Hamburg, Germany), resuspended finally in 100% methanol and 0.45 μ filtered for metabolite analysis. Samples (20 µL) were injected onto Waters 515 HPLC system connected to a photo diode array detector with C18 column maintained at ambient temperature (24–26 °C). The mobile phase was acetonitrile:water (30:70) for naringenin at 280 nm and 80% methanol for genistein detection at 260 nm with a programmed flow rate of 1.0 mL/min.

## Data Availability

The data presented in this study are available on request from the corresponding author.

## Supplementary Materials

The following are available online at https://www.mdpi.com/2223-7747/10/1/52/s1. Figure S1: Dot blot analysis of putative transformants.

## Abbreviations

BAP	Benzyl Amino Purine
CaMV	Cauliflower Mosaic Virus
cDNA	Complementary Deoxyribonucleic acid
FW	Fresh Weight
GC-MS Gas	Chromatography-Mass Spectroscopy
*GmIFS*	*Glycine max Isoflavone synthase*
HPLC	High Performance Liquid Chromatography
*IFS*	*Isoflavone synthase*
KIN	Kinetin
LB	Luria-Bertani Broth
mg L^-1^	Milligram per litre
MN	Macheray Nagel
MS	Murashige and Skoog medium
MS + B5	MS Gamborg B5 medium
NAA	Naphthalene Acetic Acid
nm	Nanometer
nM	Nanomolar
PCR	Polymerase Chain Reaction
Psi	Pound per square inch
RNA	Ribonucleic acid
rpm	Revolution per min
YENB	Yeast Extract Nutrient Broth
μg	Microgram
μL	Microlitre
μM	Micromolar

## References

[1] Agarwal R.K., Dewar H.A., Newell D.J., Das B. (1977). Controlled trial of the effect of cycloalliin on the fibrinolytic activity of venous blood. Atherosclerosis.

[2] Kalus U., Pindur G., Jung F., Mayer B., Radtke H., Bachmann K., Mrowietz C., Koscielny J., Kiesewetter H. (2000). Influence of the Onion as an Essential Ingredient of the Mediterranean Diet on Arterial Blood Pressure and Blood Fluidity. Arzneimittelforschung.

[3] Ashwini M., Sathishkumar R. (2014). Onion (*Allium cepa*)—Ethnomedicinal and Therapeutic Properties. Handbook of Medicinal Plants and Their Bioactive Compounds.

[4] Nicastro H.L., Ross S.A., Milner J.A. (2015). Garlic and onions: Their cancer prevention properties. Cancer Prev. Res..

[5] Suleria H.A.R., Butt M.S., Anjum F.M., Saeed F., Khalid N. (2015). Onion: Nature Protection Against Physiological Threats. Crit. Rev. Food Sci. Nutr..

[6] Sreevidya V., Srinivasa Rao C., Sullia S., Ladha J.K., Reddy P.M. (2006). Metabolic engineering of rice with soybean isoflavone synthase for promoting nodulation gene expression in rhizobia. J. Exp. Bot..

[7] Jung W., Yu O., Lau S.-M.C., O’Keefe D.P., Odell J., Fader G., McGonigle B. (2000). Identification and expression of isoflavone synthase, the key enzyme for biosynthesis of isoflavones in legumes. Nat. Biotechnol..

[8] Yu O., Jung W., Shi J., Croes R.A., Fader G.M., McGonigle B., Odell J.T. (2000). Production of the Isoflavones Genistein and Daidzein in Non-Legume Dicot and Monocot Tissues. Plant Physiol..

[9] Li X., Qin J.-C., Wang Q.-Y., Wu X., Lang C.-Y., Pan H.-Y., Gruber M.Y., Gao M.-J. (2011). Metabolic engineering of isoflavone genistein in Brassica napus with soybean isoflavone synthase. Plant Cell Rep..

[10] Shih C.-H., Chen Y., Wang M., Chu I.K., Lo C. (2008). Accumulation of Isoflavone Genistin in Transgenic Tomato Plants Overexpressing a Soybean Isoflavone Synthase Gene. J. Agric. Food Chem..

[11] Malla A., Srinivasan B., Shanmugaraj B.M., Ramalingam S. (2015). Micropropagation and DNA delivery studies in onion cultivars of Bellary, CO_3_. J. Crop Sci. Biotechnol..

[12] Kanaze F.I., Kokkalou E., Georgarakis M., Niopas I. (2004). Validated high-performance liquid chromatographic method utilizing solid-phase extraction for the simultaneous determination of naringenin and hesperetin in human plasma. J. Chromatogr. B.

[13] Malla A., Ramalingam S., Grumezescu A.M., Holban A.M. (2018). Chapter 11—Health Perspectives of an Isoflavonoid Genistein and its Quantification in Economically Important Plants. Role of Materials Science in Food Bioengineering.

[14] Li X.-Q., Liu C.-N., Ritchie S.W., Peng J.-Y., Gelvin S.B., Hodges T.K. (1992). Factors influencing Agrobacterium-mediated transient expression of gusA in rice. Plant Mol. Biol..

[15] Guo G., Maiwald F., Lorenzen P., Steinbiss H.-H. (1998). Factors Influencing T-DNA Transfer into Wheat and Barley Cells by Agrobacterium Tumefaciens. Cereal Res. Commun..

[16] Godwin I., Todd G., Ford-Lloyd B., Newbury H.J. (1991). The effects of acetosyringone and pH on Agrobacterium-mediated transformation vary according to plant species. Plant Cell Rep..

[17] Hooykaas P.J.J. (1989). Transformation of plant cells via Agrobacterium. Plant Mol. Biol..

[18] Rashid H., Yokoi S., Toriyama K., Hinata K. (1996). Transgenic plant production mediated by Agrobacterium in Indica rice. Plant Cell Rep..

[19] Arencibia A.D., Carmona E.R., Tellez P., Chan M.-T., Yu S.-M., Trujillo L.E., Oramas P. (1998). An efficient protocol for sugarcane (*Saccharum spp.* L.) transformation mediated by Agrobacterium tumefaciens. Transgenic Res..

[20] Khanna H., Raina S. (1999). Agrobacterium-mediated transformation of indica rice cultivars using binary and superbinary vectors. Funct. Plant Biol..

[21] Cheng M., Lowe B.A., Spencer T.M., Ye X., Armstrong C.L. (2004). Factors influencing Agrobacterium-mediated transformation of monocotyledonous species. In Vitro Cell. Dev. Biol. Plant.

[22] Hayta S., Smedley M.A., Demir S.U., Blundell R., Hinchliffe A., Atkinson N., Harwood W.A. (2019). An efficient and reproducible Agrobacterium-mediated transformation method for hexaploid wheat (*Triticum aestivum* L.). Plant Methods.

[23] Anand A., Wu E., Li Z., TeRonde S., Arling M., Lenderts B., Mutti J.S., Gordon-Kamm W., Jones T.J., Chilcoat N.D. (2019). High efficiency Agrobacterium-mediated site-specific gene integration in maize utilizing the FLP-FRT recombination system. Plant Biotechnol. J..

[24] Dönmez B.A., Dangol S., Bakhsh A. (2019). Transformation Efficiency of Five Agrobacterium Strains in Diploid and Tetraploid Potatoes. Sarhad J. Agric..

[25] Branca F., Lorenzetti S. (2005). Health Effects of Phytoestrogens. Forum Nutr..

[26] Liu C.-J., Blount J.W., Steele C.L., Dixon R.A. (2002). Bottlenecks for metabolic engineering of isoflavone glycoconjugates in Arabidopsis. Proc. Natl. Acad. Sci. USA.

[27] Liu R., Hu Y., Li J., Lin Z. (2007). Production of soybean isoflavone genistein in non-legume plants via genetically modified secondary metabolism pathway. Metab. Eng..

[28] Graham T.L. (1991). Flavonoid and isoflavonoid distribution in developing soybean seedling tissues and in seed and root exudates. Plant Physiol..

