# Supplementary material for: Metabolic Engineering of Isoflavonoid Biosynthesis by Expressing Glycine max Isoflavone Synthase in Allium cepa L. for Genistein Production
Source: Plants (Basel). 2020 Dec 29;10(1):52. doi: 10.3390/plants10010052 (PMC7823504; doi:10.3390/plants10010052)
Supplement: Supplementary file 1 [file plants-10-00052-s001.pdf]

plants

Supplementary Material

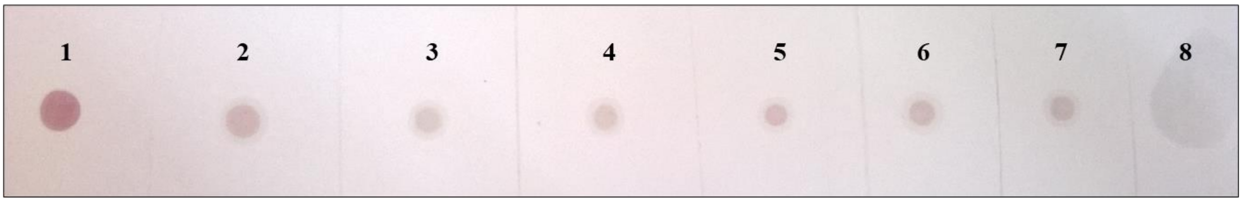

Supplementary Figure S1: Dot blot analysis of putative transformants. Lane 1, 2: Positive control (Plasmid), Lane 3-5: *Agrobacterium* transformed callus, Lane 6-7: Gene gun transformed callus, Lane 8: (Wild type callus) Negative control.
